# New method to assess the long-term role of wind energy generation in reduction of CO_2_ emissions – Case study of the European Union

**DOI:** 10.1016/j.jclepro.2018.09.249

**Published:** 2019-01-10

**Authors:** Cristina Vázquez Hernández, Javier Serrano González, Ricardo Fernández-Blanco

**Affiliations:** a European Commission, Joint Research Centre, Directorate C - Energy, Transport, and Climate, Westerduinweg 3, NL-1755, LE Petten, the Netherlands; b Group OASYS, University of Malaga, 29071, Malaga, Spain

**Keywords:** CO_2_ emissions, Wind energy, Displacement emission factor, PRIMES, Sustainability, Environmental policies

## Abstract

Most existing works using a displacement estimation method to estimate the CO_2_ emissions abated by wind energy are based on the current operating principles of the power system. They consider a fixed displacement emission factor since wind energy is assumed to replace high-carbon generation. This method may be unsuitable in the long run when the energy mix of most countries becomes more decarbonised. Consequently, wind energy would replace those technologies becoming increasingly predominant in the future, *i.e.* lower polluting fossil fuels such as natural gas and even other less competitive low-carbon technologies. In order to consider this effect, this paper proposes a new method that estimates a range of potential CO_2_ emissions abated by wind energy based on two dynamic displacement emission factors, which are periodically updated according to the evolution of the future energy mix. Such factors represent an upper and a lower limit of CO_2_ emissions avoided. The method is validated in the case study of the European Union over the period 2015–2050. The results show that the annual displacement emission factor by wind energy may vary from about 422 to 741 t CO_2_/GWh in 2015 to around 222–515 t CO_2_/GWh in 2050. The total CO_2_ abatement ranges from about 6600 to 13100 Mt CO_2_ in the period 2015–2050.

## Highlights

• A new method to estimate the potential CO_2_ abatement of wind energy is proposed.
• New dynamic displacement emission factors consider future decarbonisation of the energy mix.
• A case of study is analysed: The European Union over the period 2015–2050.
• CO_2_ abatement in Europe may range from about 6600 to 13100 Mt CO_2_ during 2015–2050.

## List of acronyms

*Acronym*: *Definition*
CO_2_: Carbon dioxide
CO_2_eq: Carbon dioxide equivalent. It represents the amount of CO_2_ which would have the equivalent global warming impact compared to any quantity and type of greenhouse gas
DAM-DEF: Dynamic all mix energy displacement emission factor
DEF: Displacement emission factor
DHC-DEF: Dynamic high-carbon displacement emission factor
EF: Emission factor
EU: European Union
EUMSs: European Union Member States
EWEA: European Wind Energy Association (currently known as WindEurope)
GHG: Greenhouse gas
GWEC: Global Wind Energy Council
HC-DEF: High-carbon displacement emission factor
IEA: International Energy Agency
IRENA: International Renewable ENergy Agency
LCA: Life cycle assessment
NG-DEF: Natural gas displacement emission factor
NREL: National Renewable Energy Laboratory of the United States of America
RES: Renewable energy sources
SEAI: Sustainable Energy Authority of Ireland
WE: Wind energy

## 1 Introduction

The European Union (EU) aims at decarbonising the energy system by 2050 by setting itself a long-term goal of reducing greenhouse gas (GHG) emissions by 80–95% compared to 1990 levels (European Commission, 2011). To meet this goal, renewable energy sources (RES) are playing an expanding role in the energy system of many EU Member States (EUMSs) and in particular, wind energy (WE) is expected to become the main contributing RES to GHG reduction. According to the strategies defined in the National Renewable Energy Action Plans, WE would account for a 43.1% of RES capacity by 2020 in the EU energy mix (Serrano González and Lacal-Arántegui, 2016). This share is expected to increase for longer horizons (2030, 2040, and 2050) although specific targets are not defined yet.

Additionally, the EUMSs are currently developing or have already developed their national laws on climate change (Reuters, 2017, Reuters, 2018, El País, 2018, Government Offices of Sweden, 2018), which reinforces the transition of their energy system towards a more decarbonised model. These national laws will be complemented by national plans of energy and climate, which will establish the measures to meet the long-term targets up to 2030 and 2050. Among these measures, a massive deployment of WE will play an essential role on reducing the GHG emissions as a consequence of a high cost reduction driven by the technology development, the competitive procurement and internationally high-experienced developers. The generation costs of onshore wind projects commissioned in 2017 largely fell within the range of fossil fuels and are expected to reach the lowest level by 2020 beating not only the fossil fuels but also the other RES (IRENA, 2018).

In this context, a proper method to estimate the potential GHG emissions^2^ averted in the long term by RES and especially WE will be of major importance so that policy makers and practitioners will be able to define a strategic energy planning with an adequate penetration of the different technologies that guarantees to meet the EU decarbonisation targets.

Estimating the actual impact of increasing WE generation on CO_2_ emissions abatement is a complex task. The current methods can be classified into four categories including the displacement estimation methods, econometric methods, methods based on dispatch and unit commitment models, and detailed simulations consisting of generation expansion models with dispatch models (Holttinen et al., 2014). The displacement estimation approach is the simplest method since WE generation is assumed to replace the average annual emissions of the power system. This method is used in the Clean Development Mechanisms to issue Certified Emissions Reductions for renewable electricity production in developing countries. As explained in Section 2, this method is also commonly used by some organisations such as WindEurope (formerly known as EWEA) and the Global Wind Energy Council (GWEC). These works consider that WE generation displaces all high-carbon generation according to a displacement emission factor (DEF) that remains constant over the years regardless of the evolution of the different technologies in the energy mix.

Such premise seems a reasonable approximation according to the current structure and operation of the electricity system: WE mostly displaces the generation units with higher marginal costs (*i.e.* natural gas- and coal-fired power plants) according to the merit-order effect that currently rules the European electricity markets. However, this assumption will need to be reformulated in the long-term as the energy mix of most countries becomes more decarbonised.

On the one hand, the price of the CO_2_ emission allowances of the EU Emissions Trading System is expected to increase considerably in the coming years (thus increasing the operating costs of carbon and gas power units). This fact, along with the planned regulatory withdrawal of coal and lignite power plants, will decrease the role of the most polluting high-carbon technologies in the energy mix so WE will replace other lower carbon intensive technologies such as natural gas. Consequently, the emission factor abated by WE will decrease over the years.

On the other hand, since RES continue to penetrate in the energy system and their costs further decrease, they will presumably change how the electricity market is ruled as concluded by Conejo and Sioshansi (2018) who recognise that “*the fundamental and underlying architecture of electric power systems are changing in major ways*” due to increasing “*weather-dependent renewable generation*”. For this reason, Qi et al. (2014) stressed the implications of assuming that RES only displace fossil fuel-based technologies. This assumption might be suitable at present but not accurate in the long run when new additions of RES capacity might displace older RES capacity.

Under these circumstances since WE penetrates in some energy systems, it is expected to increasingly displace lower polluting fossil fuels and even other less competitive RES in the long-term. As a result, the emission factor displaced by WE is expected to decrease over the years as power systems become more decarbonised. In such a context, this paper aims to answer the following research questions: How will the potential CO_2_ emissions displaced by WE differ among approaches wherein static or dynamic displacement emission factors are accounted for? What will the potential CO_2_ abatement by WE be at European (*i.e.* EU28) and national (*i.e.* EUMSs) levels up to 2050 if dynamic displacement emission factors are considered?

The displacement estimation method used in existing works (Corbetta et al., 2015, GWEC, 2016) ignores how the decarbonisation of the energy mix in the long-term in many countries will impact on the technologies displaced by WE and consequently the CO_2_ emissions avoided. Thus, the contributions of this paper are twofold:

• From a methodological viewpoint, this paper aims to fill the existing gap by proposing a new method to estimate a range of potential CO_2_ emissions abated. Such range is calculated based on two displacement emission factors which are dynamically computed on an annual basis according to the evolution of the future energy mix. The first factor considers that WE will replace future high-carbon generation providing an upper limit of potential CO_2_ emissions avoided. The second factor considers that WE will displace not only high-carbon but also other low-carbon generation resulting in a lower limit. This method clearly outperforms the displacement estimation methods used in the literature from a scientific point of view since the displacement emission factors are updated periodically. Since this method takes into account how each technology generation evolves, it enables a meaningful comparison of potential CO_2_ emissions avoided by WE across countries with high differences among their energy mixes. Furthermore, it avoids an overestimation of the CO_2_ emissions abated in those power systems that will become highly decarbonised in the long-term. Additionally, the proposed method may be extended and used to other non-EU regions given the evolution of their respective energy mixes.
• The proposed method has been implemented in the case study of the European Union by using accurate data for the evolution of the energy mix provided by the *EU Reference Scenario 2016* (European Commission, 2016) by the PRIMES model (E3Mlab, 2006). As a result, this paper presents an exhaustive study of the potential CO_2_ emissions avoided by WE in the EUMSs in the long-term. This estimation is a fundamental aspect to be considered by policy officers, when developing the climate change laws and the national energy and climate plans to meet the long-term GHG targets. To the best of the authors' knowledge, this is the first manuscript estimating the potential CO_2_ emissions avoided by WE in the EU by using a dynamic displacement estimation approach.

The remainder of this paper is organised as follows: Section 2 presents the literature review, Section 3 introduces the new displacement estimation method proposed to assess the role of WE in reducing CO_2_ emissions as well as the different approaches analysed to validate the method. Section 4 presents the input data of a case study for the EU and thoroughly discusses the numerical results. Finally, the conclusions are drawn in Section 5.

## 2 Literature review

The assessment of GHG from different human activities is a research area covering fields as diverse as agricultural processes (Chareonpanich et al., 2017, García-Gusano et al., 2015), industrial applications (Nabavi-Pelesaraei et al., 2014, Raucci et al., 2015) as well as transport (Hoppe et al., 2016, Robèrt et al., 2017) and energy sectors (Marchi et al., 2018, Weldemichael and Assefa, 2016, Wesseh and Lin, 2015).

Within the energy sector, the role of WE and other RES in the decarbonisation of the energy system has been analysed in the scientific literature throughout two main approaches: (i) life cycle assessment (LCA) in order to quantify the overall GHG emissions by RES during their life span (Dolan and Heath, 2012, NREL, 2013a, Amponsah et al., 2014, Nugent and Sovacool, 2014, Asdrubali et al., 2015) and (ii) RES potential for CO_2_ abatement by replacing the most pollutant energy sources. This paper focuses on the CO_2_ abatement by WE and the methods to estimate it can be categorised into fourfold. Even though the interested reader is referred to (Holttinen et al., 2014) for a detailed explanation of each category, the methods are briefly explained next:

• **Displacement estimation methods.** They assume that wind energy replaces the average annual emission of other technologies of the energy mix. Although it may lead to inaccuracies due to their simplifying assumptions, it is characterised as a straightforward, understandable, and computationally inexpensive approach to estimate CO_2_ emissions abated by WE. Little attention has been paid to these methods (Corbetta et al., 2015, GWEC, 2016, SEAI, 2017).
• **Econometric methods.** Statistical tools are used to estimate CO_2_ emissions based on historical data on variables which are highly related to these emissions. However, these methods may overlook that the CO_2_ emissions are nonlinear and very sensitive to the data. These methods have been applied in (Wheatley, 2013, Cullen, 2013, Amor et al., 2014, Thomson et al., 2017).
• **Methods based on dispatch and unit commitment models.** These are more sophisticated and optimisation-based approaches to compute the CO_2_ emissions abated by WE. Their goal is to optimise the dispatch and commitment of the generating units driven by the generation cost minimisation and taken into account a set of technical and physical constraints while satisfying the energy demand. They are able to capture the particular system conditions given a time horizon and an energy mix. However, running daily simulations with hourly time steps for several future years may be computationally expensive. Works based on these methods are (Holttinen and Tuhkanen, 2004, Delarue et al., 2009, Valentino et al., 2012, NREL, 2013b).
• **Detailed simulations with generation expansion and dispatch-based methods.** This approach requires to optimise the generation energy mix by using generation expansion models before running the economic dispatch to compute the CO_2_ emissions abated by WE. This method is deemed the more sophisticated one but the most computationally expensive. For instance, Kiviluoma and Meibom (2010) implemented such method by combining the electricity and district sectors. The authors presented a detailed analysis of the flexibility of the power system, CO_2_ emissions, and costs for different scenarios; however, they recognised the sensitivity of the results with the input data.

EWEA and GWEC have assessed the potential CO_2_ emissions abatement by WE up to 2030 and 2050, respectively. In particular, the assessment performed by EWEA (Corbetta et al., 2015) has been at European level and by GWEC (GWEC, 2016) at global level. Even though neither of these reports provides a detailed description of the method used, it may be inferred that the results were obtained considering the long-term scenarios for WE penetration provided in each respective report and assuming a fixed DEF per unit of energy generated by WE. By performing simple calculations (*i.e.* dividing CO_2_ savings by the estimated electricity generated by WE in the long-term scenarios) based on the data given by both reports, a constant DEF of 560 g CO_2_/kWh can be deduced from the EWEA report and a similar constant DEF of 600 g CO_2_/kWh from the GWEC report. Based on the order of magnitude of these values, the potential CO_2_ emissions avoided by WE are calculated by assuming that it replaces high-carbon technologies.

Holttinen and Tuhkanen (2004) presented a case study about the effect of wind power on CO_2_ reduction in the Nordic countries. The Nordic electricity market was simulated with and without wind production using the power market model EMPS, which is a commercial model developed at SINTEF Energy Research. The paper revealed that initially wind power would replace mostly coal-fired units, which would lead to a reduction of 620–700 g CO_2_/kWh. However, the authors also raised an alternative scenario in which WE mostly replaced natural gas combined-cycle power plants achieving a considerably lower CO_2_ reduction of about 300 g CO_2_/kWh.

Gil and Joos (2007) presented a short-term generalised method considering typical system dispatch strategies and comparing real WE generation profiles with the evolution of the marginal emissions, *i.e.* a hybrid between the displacement estimation and econometric methods. The authors stated that the main objective of their work was to contribute to the development of simplified methodologies to assess the displaced emissions by renewable energy projects.

In the same line of research, Delarue et al. (2009) presented a method to assess the actual effect of WE on CO_2_ emissions and electricity generation costs based on unit commitment model by taking into consideration several technical constraints and the effect of wind power imbalances on the electrical system. The simulation carried out over a case study for Belgium showed that CO_2_ emissions reduction would reach a level of 1.26 kt CO_2_ avoided per year for each MW of new wind power capacity installed.

Valentino et al. (2012) studied the implications from the power system operation in the state of Illinois (United States) in 2006 with increasing levels of wind energy. The analysis relied on the results from a dispatch and unit commitment model by accurately modelling the cycling and start-ups of fossil-fuel power plants. The CO_2_ emissions avoided by WE decreased up to 33% for the case of 40% of WE penetration with respect to the case without WE penetration.

Wheatley (2013) devised an empirical approach to compute emissions savings by using historical data from the Irish grid such as the electricity demand, wind generation and detailed characteristics of the generating units. This paper concluded that the emissions displaced by WE were 0.28 t CO_2_/MWh for this system. In the same vein, Cullen (2013) used another empirical method to estimate the emissions avoided on the Texas electricity grid by WE between 2005 and 2007. The dynamics of fossil-fuel generators were captured by using demand, wind power, temperature, and network congestion data, as well as their corresponding lagged values.

NREL (2013b) conducted a system-wide analysis of the Western Interconnection of the United States by using a dispatch-based model. Among other metrics, the report concentrated on the GHG emissions avoided by RES. For instance, this work reported that the Western grid would avoid 29–34% CO_2_ emissions when the wind and solar energy penetration in that portion of the United States is up to 33% compared to the base case. According to Holttinen et al. (2014), this can be translated into 0.489–0.523 t CO_2_/MWh of wind/solar generation approximately.

Amor et al. (2014) examined the impacts of WE generation on electricity prices and GHG emissions for the Ontario electricity grid (Canada) during six years (2006–2011). The authors used an econometric model to account for the internal grid congestion of the electricity system of Ontario.

Qi et al. (2014) presented a detailed analysis on the impact of RES development on CO_2_ emissions in China by using a multi-regional, multi-sectoral, equilibrium model. The authors used the Current Policy scenarios obtained by the China-in-Global Energy Model showing three levels of penetration (low, medium and high) for RES over the period 2010–2050. This paper stressed the implications of assuming that RES only displace fossil fuel-based technologies, as done in most existing works in the literature. This assumption might be suitable at present but not accurate in the long run when new additions of RES capacity might displace older RES capacity. Two approaches were compared: the “ideal” approach in which new additions of RES only displace fossil fuel-based generators and a second approach that considers economy-wide effects (considering the effect of implemented policies on energy prices as well as the transmission effect across local and global markets). The results showed that the realistic approach gives a CO_2_ annual abatement level from 17 to 35% lower than the “ideal” approach by 2050.

Thomson et al. (2017) presented a marginal displacement approach considering a half-hourly sampling in order to assess the GHG emissions abated by WE during the period 2009–2014 in Great Britain. The results showed an evolution of the GHG emissions displacement from 1.99 ± 0.22 Mt CO_2_eq in 2009 to 10.21 ± 0.29 Mt CO_2_eq in 2014.

Usubiaga et al. (2017) presented a historical scenario-based analysis for individual renewable energy technologies for the period 1990–2013. The proposed method consisted of defining alternative counterfactual scenarios to analyse what could have happened if each one of the renewables technologies under study (solar photovoltaic, onshore and offshore wind energy) had not been deployed in the European power system. The results showed that onshore wind had a virtually net contribution to emissions reduction from 1990, whilst offshore wind has a net positive contribution to CO_2_ emissions since 2004.

In 2017, the SEAI estimated the fuel displacement from renewable electricity generation in Ireland using a displacement estimation method known as Primary Energy Equivalent approach. The results obtained were further refined by means of a detailed dispatch model of the operation of the entire all-island electricity system in the year 2012, so that the effects of ramping and cycling of fossil fuel plants were accounted for. The estimated amount of CO_2_ avoided from RES reached 3932 kt CO_2_ in 2016 of which 2188 kt CO_2_ were displaced by WE (SEAI, 2017). Based on wind energy generation in 2016 in Ireland, the displacement emission factor by this technology in Ireland was 355 g CO_2_/kWh.

This paper provides an added value over the technical literature presented above for two reasons: (i) little attention has been paid to the displacement estimation methods, and (ii) the displacement estimation methods used by wind industry associations (Corbetta et al., 2015, GWEC, 2016) are based on static displacement emission factors. In addition to the scientific contribution, this paper also intends to support policy makers in the development of new regulatory frameworks aimed at achieving the GHGs emission reduction targets in the long-term by providing a novel scientific and exhaustive analysis on the actual contribution of WE to the decarbonisation of the European electricity system.

## 3 Method

The method proposed in this paper assumes that the gross electricity generated by WE in the year *y* displaces the electricity generated by other technologies according to their presence in the energy mix in the same year. Thus, the displacement emission factor is dynamic, *i.e.* it evolves over the years as the generation of the technologies in the energy mix does.

### 3.1 Notation

The variables, parameters, sets and indices defined to estimate the CO_2_ emissions displaced by WE generation are displayed in Table 1.

**Table 1:** Variables, parameters, sets and indices used in the proposed method.

Type	Symbol	Definition
Index	*t*	Index of technology $\left(t\;\in T_{y}\right)$
Index	*y*	Index of year (y∈Y)
Index	*c*	Index of country (c∈C)
Set	*T*_*y*_	Set of technologies displaced in year *y*
Parameter	*EF*_*t*_	Emission factor of technology *t*
Parameter	*N*_*y*_	Number of years
Parameter	*N*_*c*_	Number of countries
Variable	$E_{Wind,y}^{AGG}$	Aggregated gross electricity generation by wind for the set of countries under study and year *y*
Variable	$DEF_{y}^{AGG}$	Aggregated displacement emission factor for the set of countries under study and year *y*
Variable	*E*_*t,c,y*_	Gross electricity generation by technology *t* in country *c* and year *y*

### 3.2 Dynamic displacement estimation approach

The proposed method aims at estimating the annual and cumulative CO_2_ abatement potential of WE. The region under study can be composed by several sub-regions such as countries or markets. For this reason, a bottom-up method is proposed so that the contribution of WE to CO_2_ reduction is individually calculated for each sub-region (in order to take into account the internal energy mix of each country or market) and for every year (in order to consider the changes in the generation structure over the years).

The necessary inputs to implement the method are (i) the scenarios of annual energy generated by each technology in each sub-region or country considered over the period analysed and (ii) the emission factor of each generation technology. As shown later in Section 4, the case study presented in this paper considers the long-term generation scenario known as *EU Reference Scenario 2016* from the PRIMES model as well as different emission factors collected from the literature.

**Fig. 1:**
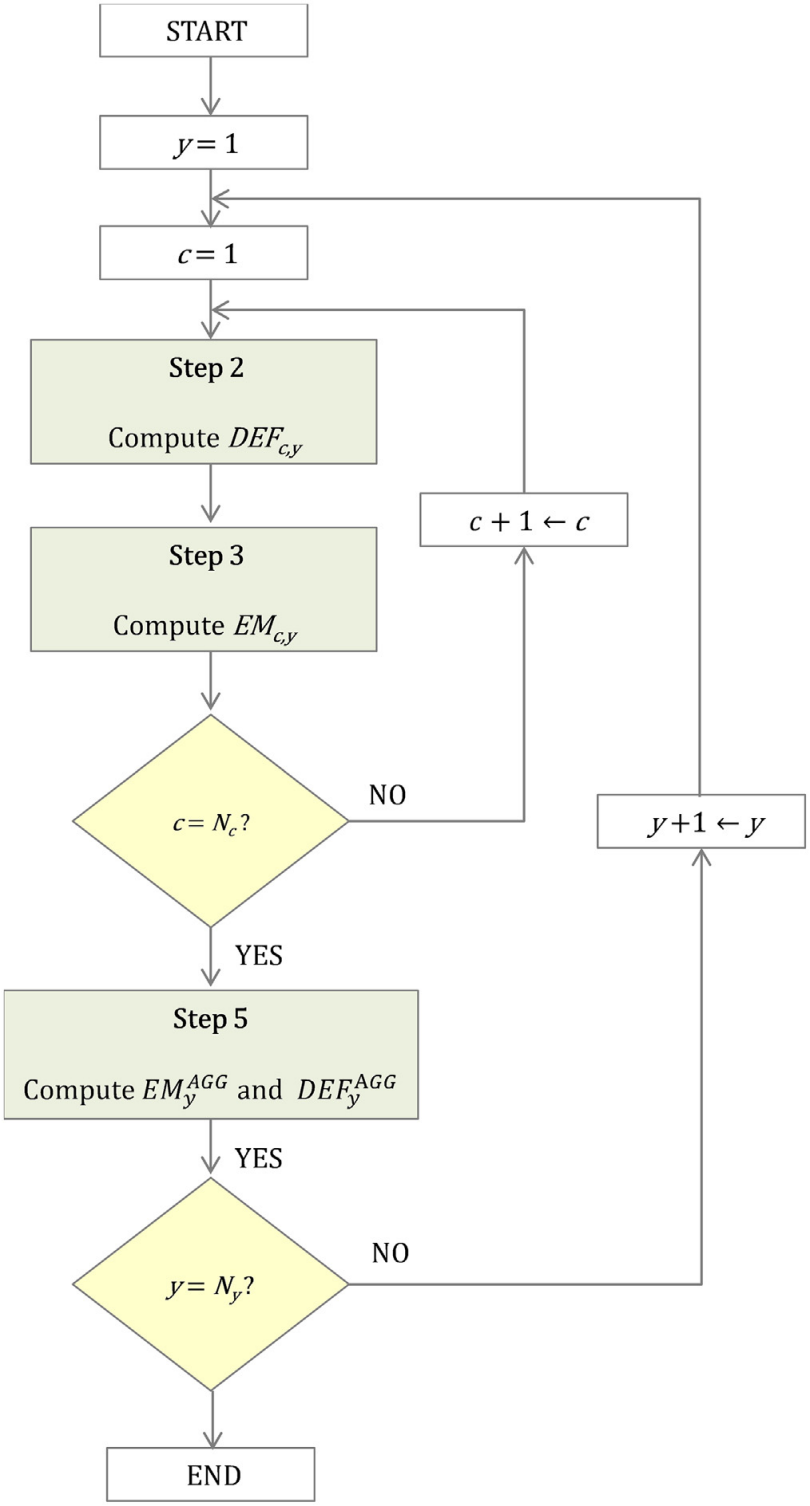
Flowchart of the proposed method to estimate the potential CO_2_ emissions abated by WE at aggregated level based on a dynamic displacement emission factor.

Fig. 1 shows the flowchart of the proposed method. As WE generation replaces other technologies based on their presence in the internal energy mix of each sub-region every year, the displacement emission factor, *DEF*_*c,y*_, must be calculated every year, *y*, in each country, *c*, with a different energy mix. The total aggregated CO_2_ emissions displaced for the set of countries under study, $EM_{y}^{AGG}$, are the summation of the total CO_2_ emissions displaced at country level, *EM*_*c,y*_.

The calculation steps to estimate the potential CO_2_ emissions abated by WE at aggregated country level are described as follows:

**Step 1**: initialisation of the country, *c*, and year, *y*, indices.

**Step 2**: the displacement emission factor, *DEF*_*c,y,*_ in country, *c*, and year, *y*, is computed according to the following equation:

(1) $$DEF_{c,y}=\frac{\sum_{t\in T_{y}\setminus Wind}E_{t,c,y}\cdot EF_{t}}{\sum_{t\in T_{y}\setminus Wind}E_{t,c,y}}\;\forall \;y\in N_{y}\;,\;\forall \;c\in N_{c}$$

Where *T*_*y*_*∖Wind* stands for those technologies that are assumed to be displaced by WE generation. It is worth noting that the displacement emission factor, *DEF*_*c,y,*_ will depend on different assumptions, which are analysed by the four different approaches described later in Section 3.3.

**Step 3**: the CO_2_ emissions avoided by wind energy, *EM*_*c,y*_, in country, *c,* and year, *y,* are computed according to the following equation:

(2) $$EM_{c,y}=E_{Wind,c,y}\cdot DEF_{c,y}\;\forall \;y\;\in N_{y},\;\forall \;c\in N_{c}$$

**Step 4**: if the country index, *c*, is not equal to the number of countries, *N*_*c*_, the calculation procedure goes to step 2. Otherwise, it goes to step 5.

**Step 5**: the aggregated CO_2_ emissions avoided, $EM_{y}^{AGG}$, for the set of countries under study in year, *y,* are computed according to the following equation:

(3) $$EM_{y}^{AGG}=\sum_{c\;\in \;N_{c}}EM_{c,y}=\sum_{c\;\in \;N_{c}}E_{Wind,c,y}\cdot DEF_{c,y}\;\forall \;y\;\in N_{y},\;\forall \;c\in N_{c}$$

The equation (3) can also be expressed as follows:

(4) $$EM_{y}^{AGG}=E_{Wind,y}^{AGG}\cdot DEF_{y}^{AGG}\;\forall \;y\;\in N_{y},\;\forall \;c\in N_{c}$$

The aggregated displacement emission factor, $DEF_{y}^{AGG}$, for the set of countries under study in year, *y*, is calculated by equating the equations (3), (4) as follows:

(5) $$DEF_{y}^{AGG}=\frac{\sum_{c\;\in \;N_{c}\setminus Wind}E_{Wind,c,y}\cdot DEF_{c,y}}{E_{Wind,y}^{AGG}}\;\forall \;y\;\in N_{y},\;\forall \;c\in N_{c}$$

**Step 6:** if the year index, *y*, is not equal to the number of years, *N*_*y*_, the calculation procedure goes to step 2. Otherwise, the procedure stops.

### 3.3 Approaches analysed

As stated in the introduction, the method commonly used in the literature to estimate the CO_2_ abatement potential of WE is based on the operating margin of the power system. In the electricity markets, WE usually displaces high-carbon technologies according to the dispatch and schedule. Thus, the potential CO_2_ emissions displaced are commonly estimated based on a static/fixed DEF corresponding to either natural gas or all high-carbon generation in the current energy mix.

These approaches are appropriate in the current scenario but they may lead to an inadequate assessment of CO_2_ emissions abated by WE in the long-term. The future composition of the energy mix of most countries will vary considerably. On the one hand, the share of high-carbon technologies in the energy mix is expected to become greatly lower. In addition, they will evolve from high polluting fossil fuels (mainly coal and lignite) to natural gas. Thus, WE is expected to replace high-carbon technologies in a different proportion compared to the current situation. On the other hand, the renewable energy generation will become more and more dominant so wind energy could displace not only high-carbon but also other renewable energy generation from less competitive sources.

These assumptions are taken into account by analysing two approaches according to the method proposed in Section 3.2 as follows:

• *Dynamic High-Carbon Displacement Emission Factor approach* (DHC-DEF approach). This approach assumes that WE will only replace high-carbon technologies (*i.e.* coal and lignite, petroleum products, natural gas and coke & blast-furnace gasses according to the categorisation by fuel type in the *EU Reference Scenario* 2016 analysed in the case study presented in Section 4). The DEF will be computed annually according to the evolution of the gross electricity generation of high-carbon technologies in the energy mix.
• *Dynamic All Mix Energy Displacement Emission Factor approach* (DAM-DEF approach). It considers that WE will replace all technologies of the energy mix. As in the DHC-DEF approach, the DEF will be dynamically computed on an annual basis according to the evolution of the gross electricity generation of all technologies.

The DHC-DEF and DAM-DEF approaches are proposed with a twofold purpose: (i) to quantify the possible effects of the long-term changes in the energy mix and the structure of the electricity markets in the estimation of the total CO_2_ abatement by WE as well as (ii) to provide a maximum level of CO_2_ emissions avoided by WE (corresponding to the DHC-DEF approach) and a minimum level in the long-term (corresponding to the DAM-DEF).

In addition, these approaches will be compared in the case study presented in Section 4 with the two approaches used in the existing literature:

• *Natural Gas Displacement Emission Factor approach* (NG-DEF approach). It assumes that WE will only replace natural gas generation so the DEF has a constant value of 405 t CO_2_/GWh over the years (International Energy Agency, 2015).
• *High-Carbon Displacement Emission Factor approach* (HC-DEF approach). This approach is based on the assumption that WE will only displace high-carbon technologies according to their current share in the energy mix. Thus, a constant DEF is considered equal to 560 t CO_2_/GWh, similarly to the value used by WindEurope (Corbetta et al., 2015).

## 4 Results and discussion

The proposed method estimates the potential CO_2_ emissions abated by WE in the EU28 over the period 2015–2050. As shown in Section 3, the potential CO_2_ emissions abated at aggregated level (*i.e.* EU) must be first computed at EUMS level. Sections 4.1 and 4.2 present the data used in this case study including the evolution of the EU energy mix in the long-term and the emission factors of each technology. Sections 4.3 and 4.4 present and discuss the numerical results. Firstly, the method proposed is validated at EU level by comparing the different approaches in Section 4.3. Secondly, the method estimates a range of potential CO_2_ emissions abated by WE in the EUMSs over the period 2015–2050 in Section 4.4. Finally, Section 4.5 thoroughly discusses the numerical results.

### 4.1 Evolution of the EU energy mix in the long-term

The *EU Reference Scenario 2016* provided by the PRIMES model (European Commission, 2016) has been considered as evolution of the energy mix in the EU28. This scenario estimates the evolution of the power capacity installed and the electricity generation by both plant and source type in the EU up to 2050.

PRIMES is an energy system modelling tool, which simulates a multi-market equilibrium solution for demand and energy supply. This model is structured for different energy sectors and simulates the European energy system and markets on a country-by-country basis. The model considers input data from Eurostat statistics and deals with different assumptions regarding several drivers such as economic development, technology evolution and other policy targets or constraints, among others. PRIMES is widely used by the European Commission and national governments to analyse the impact of environmental and energy policy instruments within each one of the 28 EUMSs. As an output, the model provides detailed projections of technology provision up to 2050, in 5-year intervals (E3Mlab, 2006). Note that the values corresponding to the intermediate years are estimated by linear interpolation.

For the sake of simplicity, the following lines briefly describe the most important figures about installed power capacity and gross electricity generation by the different technologies at EU level (although, as shown in Section 3, the proposed methodology is based on a country level analysis).

**Fig. 2:**
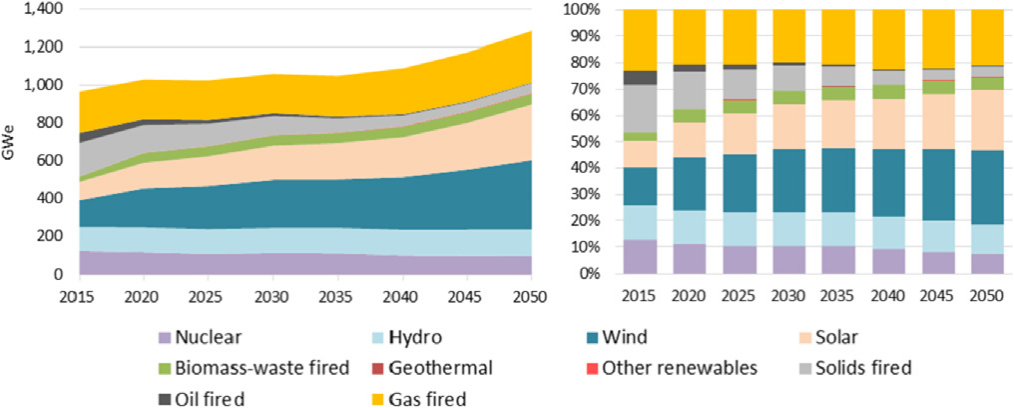
Net installed power capacity by plant type in the EU28 in the period 2015–2050. Source: Based on *EU Reference Scenario 2016*-PRIMES model.

The *EU Reference Scenario 2016* provides the evolution of the net installed power capacity by plant type in the EU as shown in Fig. 2. Total power capacity evolves from about 966 GW in 2015 to 1283 GW by 2050, which represents an increase of more than 33% of capacity installed in the European Union. Renewable energy capacity increases by 120% from 395 GW in 2015 (41% of total net installed power capacity) to about 866 GW (67%) in 2050. On the contrary, the high-carbon net installed power capacity decreases by 28% from almost 450 GW in 2015 to 325 GW in 2050 as a result of a reduction of both solids and oil-fired power plants that exceeds the net increase of gas-fired power plants (mainly natural gas) in the period 2015–2050.

Wind power capacity could increase by 1.6 times from 142 GW in 2015 to about 368 GW by 2050. The net installed wind power potential would represent 42% of renewable power and 29% of total installed power installations in 2050 compared to 36% and 15% in 2015 respectively.

**Fig. 3:**
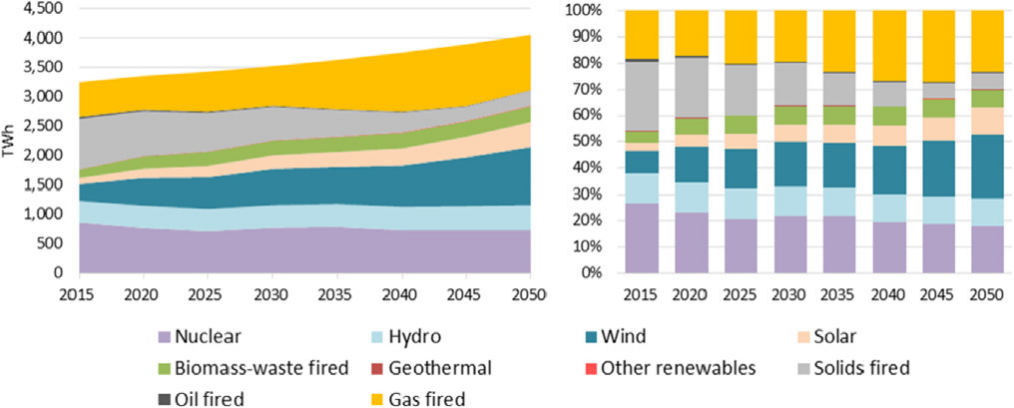
Gross electricity generation by plant type in the EU28 2015–2050. Source: Based on *EU Reference Scenario 2016*-PRIMES model.

The *EU Reference Scenario 2016* also presents an evolution of the EU gross electricity generation by plant type in the long-term to meet the energy and emissions targets in 2020 and 2030 as shown in Fig. 3.

In order to perform a more accurate estimation of the potential CO_2_ emissions abated, we have considered the gross electricity generation by fuel type. Table 2 displays such data at aggregated level in the EU. Nevertheless, it is worth noting that, as described in Section 3, the proposed methodology also considers the annual gross electricity generation by fuel type disaggregated at country level in order to compute *DEF*_*c,y*_ and *EM*_*c,y*_ (Equations (1) and (2), respectively).

**Table 2:** Gross electricity generation by fuel type in the EU28 over the period 2015–2050.

	2015	2020	2025	2030	2035	2040	2045	2050
**Gross Electricity generation by fuel type (TWh)**	**3251.3**	**3357.7**	**3430.6**	**3527.5**	**3632.8**	**3759.8**	**3900.1**	**4063.7**
Nuclear energy	867.4	773.0	717.7	777.7	789.9	734.1	732.4	736.5
Renewables	936.4	1214.6	1354.2	1512.8	1590.6	1757.0	1978.7	2235.3
Hydro	362.4	375.6	375.5	379.0	386.8	395.4	408.3	421.1
Wind	274.3	462.7	527.4	608.5	619.2	691.9	821.5	980.0
Solar, tidal etc.	104.3	155.7	194.1	234.4	259.8	298.7	360.4	435.8
Biomass & waste	188.8	213.1	249.7	283.5	317.4	363.5	381.5	391.4
Geothermal heat	6.6	7.5	7.5	7.5	7.5	7.5	7.0	7.0
Fossil fuels	1447.5	1370.1	1358.7	1237.0	1252.3	1268.7	1189.0	1091.9
Coal and lignite	846.8	767.3	655.4	562.7	441.5	329.4	231.3	251.5
Petroleum products	34.6	21.8	21.3	19.3	14.2	14.0	12.2	4.8
Natural gas	537.5	554.6	651.9	629.7	774.1	905.7	927.3	818.7
Coke & blast-furnace gasses	28.6	26.4	30.2	25.2	22.4	19.7	18.1	16.8
Other fuels (hydrogen, methanol)	0.0	0.0	0.0	0.0	0.0	0.0	0.0	0.0

Source: EU Reference Scenario 2016-PRIMES model.

Note: The *EU Reference Scenario 2016*-PRIMES model has not released the gross electricity generation by fuel type at EUMS level so such breakdown is not shown in this paper.

The *EU Reference Scenario 2016* clearly shows how the EU electricity mix moves away from coal and lignite to renewable energy sources and natural gas with the aim of meeting the energy and emissions targets for 2020 and 2030.

Total electricity generation reaches about 4065 TWh in 2050 representing 25% higher than 2015 level. The share of renewables in the total net electricity generation is expected to grow: In 2015 about 936 TWh represented 29% of the EU total gross electricity generation increasing in the long-term to cover 55% in 2050 (about 2235 TWh).

In this context, wind generation could reach 980 TWh in 2050 representing an increase of 257% compared to 2015 levels. The share of WE in the total gross electricity generation is also expected to grow from 8% in 2015 to 24% in 2050. Its share within the renewable generation will also increase from 29% in 2015 to 44% by 2050.

**Fig. 4:**
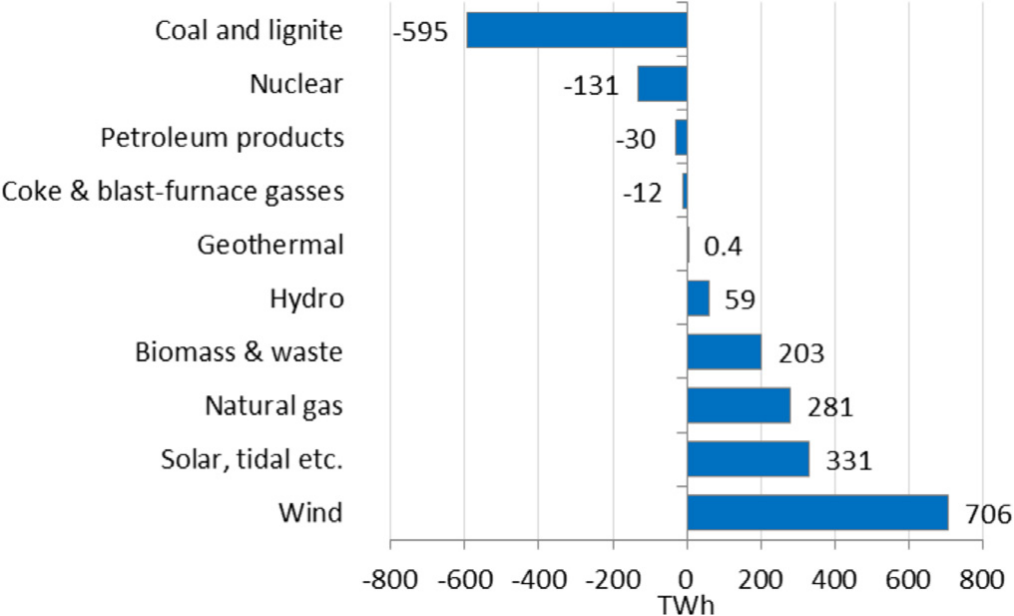
Net cumulative variation of gross electricity generation by fuel type in the EU28 over the period 2015–2050. Source: Based on *EU Reference Scenario 2016*-PRIMES model.

As shown in Fig. 4, WE shows the highest growth (about 700 TWh) in the electricity generation between 2015 and 2050 compared to other generation sources, far followed by solar and tidal, natural gas and biomass and waste. Hydro and geothermal gross electricity generation also increase in the period 2015–2050, albeit to a lesser extent. On the contrary, high-carbon gross electricity generation would decrease by 2050, mainly because of a significant fall of coal and lignite generation of almost 600 TWh and despite the increase of natural gas generation.

### 4.2 Emission factors of technologies

**Fig. 5:**
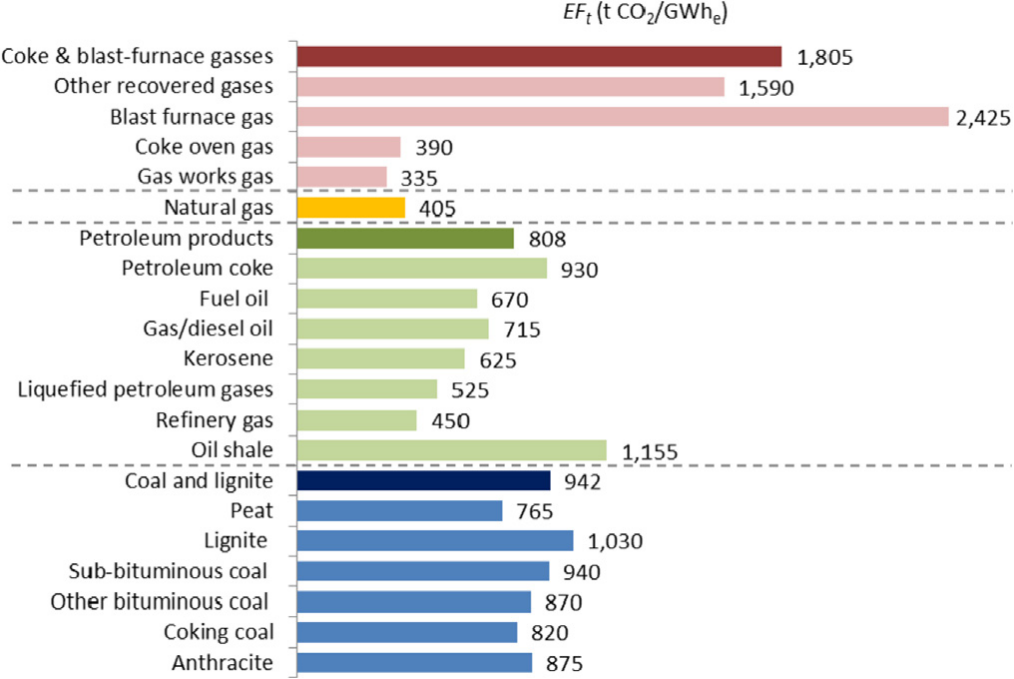
*EF*_*t*_ for high-carbon technologies considered in this work.

The *EF*_*t*_ for each high-carbon technology by fuel type is shown in Fig. 5. The emission factors of these technologies correspond to their electricity production process whilst upstream and downstream processes are rejected.

As shown in Table 3, the *EF*_*t*_ of high-carbon technologies has been taken from the report *World CO*_*2*_ *emissions from fuel combustion. Database documentation* published by the International Energy Agency (2015). Since the classification by fuel type provided by the International Energy Agency (IEA) is more disaggregated than the *EU Reference Scenario 2016*, some emission factors have been estimated. As an example, the fuel type “coal and lignite” in the *EU Reference Scenario 2016* may include different types of coal such as anthracite, sub-bituminous coal, lignite or peat whose *EF*_*t*_ values are provided by the IEA.

**Table 3:** Source of *EF*_*t*_ for high-carbon technologies.

Fuel type	*EF*_*t*_ (t CO_2_/GWh_e_)	Source
Coal and lignite	942	Estimated as weighted average of the EFs of anthracite, coking coal, other bituminous coal, sub-bituminous coal, lignite and peat based on the share of gross electricity generation of these fuels in 2015 according to Eurostat data (Eurostat, 2015). The EF of each fuel is based on (International Energy Agency, 2015)
Petroleum products	808	Estimated as weighted average of the EFs of oil shale, refinery gas, liquefied petroleum gases, kerosene, gas/diesel oil, fuel oil and petroleum coke based on the share of gross electricity generation of these fuels in 2015 according to Eurostat data (Eurostat, 2015.). The EF of each fuel is based on (International Energy Agency, 2015)
Natural gas	405	IEA (International Energy Agency, 2015)
Coke & blast-furnace gasses	1805	Estimated as weighted average of the EFs of gas works gas, coke oven gas, blast furnace gas and other recovered gases based on the share of gross electricity generation of these fuels in 2015 according to Eurostat data (Eurostat, 2015). The EF of each fuel is based on (International Energy Agency, 2015)

**Fig. 6:**
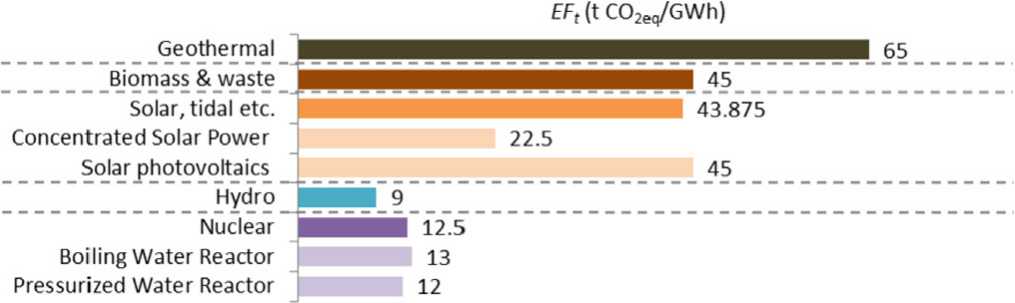
*EF*_*t*_ for renewable energy and nuclear technologies considered in this work.

Renewable energy and nuclear technologies do not emit CO_2_ during its operation process. As shown in Fig. 6, the *EF*_*t*_ corresponding to their lifecycle GHG emissions (t CO_2_eq/GWh) is negligible however it has been considered in the calculations in order to produce more accurate numerical results. The *EF*_*t*_ of the renewable energy and nuclear technologies has been taken from the report *Life Cycle Assessment Harmonization* (NREL, 2013a) performed by the National Renewable Energy Laboratory of the United States as displayed in Table 4.

**Table 4:** Source of *EF*_*t*_ for renewable energy and nuclear technologies.

Fuel type	*EF*_*t*_ (t CO_2_eq/GWh)	Source
Nuclear	13	Estimated as weighted average of the EFs of Pressurized Water Reactor and Boiling Water Reactor based on the share of gross electricity generation of these fuels in 2015 according to Eurostat data (Eurostat, 2015). The EF of each fuel is based on (NREL, 2013a)
Hydro	9	NREL (NREL, 2013a)
Solar, tidal, etc.	44	Only solar energy is considered under this fuel type. The *EF*_*t*_ is estimated as the weighted average of the EFs of Solar photovoltaics and Concentrated Solar Power based on the share of gross electricity generation of these technologies in 2015 according to Eurostat data (Eurostat, 2015). The EF of each fuel is based on (NREL, 2013a)
Biomass & waste	45	NREL (NREL, 2013a)
Geothermal	65	NREL (NREL, 2013a)

Note that all emission factors *EF*_*t*_ are considered constant values in the equation (1). Even though some technological improvements may arise in the operational process of high-carbon technologies or in the upstream and downstream processes of RES, there is a relatively small room for improvement of their *EF*_*t*_ in the long-term.

### 4.3 Validation of the method at EU level

This section aims at comparing the results from the approaches proposed, which dynamically update the displacement emission factor on an annual basis, with the results from the approaches used in the technical literature, which are based on a static displacement emission factor. This can be viewed as a sensitivity analysis of the displacement emission factor.

**Fig. 7:**
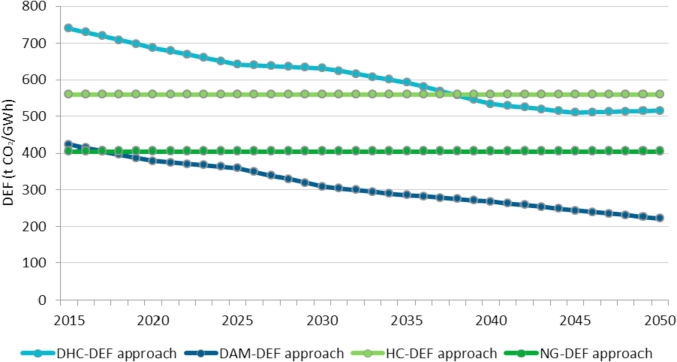
Evolution of the DEF in the EU over the period 2015–2050 under different approaches.

Fig. 7 shows the evolution of the DEF in each approach over the analysed period. As mentioned in Section 3.3, the DEF remains constant in the approaches currently used in the literature, *i.e.* WE is assumed to replace either natural gas generation only (NG-DEF) or high-carbon generation according to their share in the current energy mix (HC-DEF). On the contrary, the DEF decreases over time in the two new approaches proposed since it is presumed that WE will replace high-carbon technologies (DHC-DEF) or all mix of technologies (DAM-DEF) according to their evolution in the energy mix. The DEF in the DHC-DEF approach decreases by 31% from 741 to 515 t CO_2_/GWh over the period 2015–2050 as a result from (i) the decreasing electricity generation by the most polluting high-carbon technologies (*i.e.* coal and lignite, petroleum products and coke & blast-furnace gasses) and (ii) the increasing natural gas generation with a lower EF. In the DAM-DEF approach, the decline of the DEF reaches 47% in the same period from 422 to 222 t CO_2_/GWh. This greater fall is due to the additional displacement of renewable and nuclear electricity generation.

**Fig. 8:**
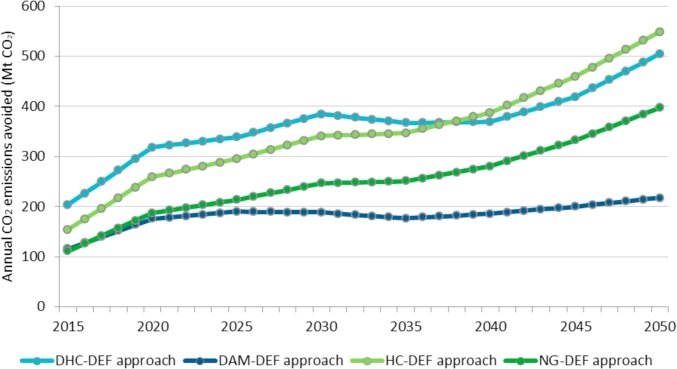
Annual CO_2_ emissions avoided by WE generation in the EU over the period 2015–2050 under different approaches.

**Fig. 9:**
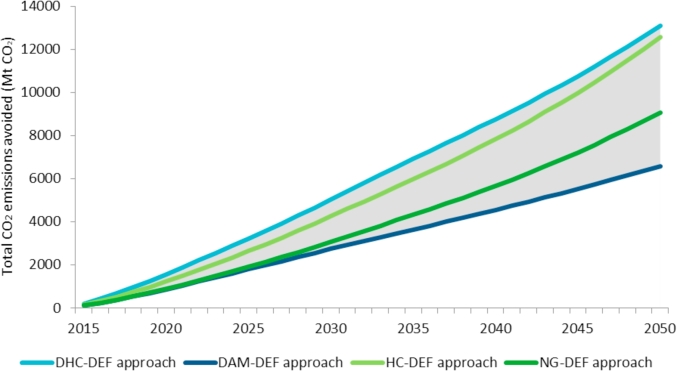
Total cumulative CO_2_ emissions avoided by WE generation in the EU over the period 2015–2050 under different approaches.

The potential annual and cumulative CO_2_ emissions avoided by WE in the EU calculated with all approaches are presented in Fig. 8, Fig. 9.

From the results shown in Fig. 8, Fig. 9, the following remarks can be drawn:

• The NG-DEF approach — WE replaces natural gas only — is a simple method since the constant DEF does not take into account how the energy mix evolves. As WE electricity penetration increases, the potential CO_2_ emissions avoided grow proportionally. The annual CO_2_ emissions abated show a 3.6-fold increase from 111 Mt in 2015 to 397 Mt in 2050. The total CO_2_ emissions avoided reach 9080 Mt over the period 2015–2050. As a reference, this represents almost three times the annual emissions of the energy sector in the EU (according to the European Environmental Agency, these CO_2_ emissions reached around 3240 Mt CO_2_ in 2015).
• As the previous case, in the HC-DEF approach — WE replaces a constant high-carbon electricity mix over the years — the DEF remains constant over time so the potential CO_2_ emissions abated are proportional to the growth of the WE generation only. Nevertheless, a higher DEF compared to the NG-DEF approach results in a higher CO_2_ abatement potential. The annual CO_2_ emissions avoided also increase to 3.6 folds from 154 Mt in 2015 to 549 Mt in 2050. This figure is similar to 554 Mt CO_2_ estimated by WindEurope in 2030 as a result of 988.3 TWh of WE penetration in their High Scenario (Corbetta et al., 2015). The total CO_2_ emissions avoided reach 12554 Mt over the period 2015–2050. WindEurope does not estimate cumulative CO_2_ emissions displaced.
• The DHC-DEF approach — WE replaces a dynamic high-carbon energy mix over the years — estimates a DEF which evolves as the high-carbon generation does. Up to 2030, the annual CO_2_ emissions avoided display an upward trend from 203 Mt in 2015 to 385 Mt in 2030. These are the highest values compared to the other approaches since the high polluting fossil fuels represent a high share in the energy mix of most EUMSs over this period. On the contrary, in the period 2030–2035 the annual CO_2_ emissions show a slight decrease from 385 to 367 Mt as a consequence of the drop in the coal and lignite share in the energy mix. From 2035 onwards, the annual CO_2_ emissions avoided recover an upward trend reaching 505 Mt in 2015 mainly because of the increasing natural gas generation. The total CO_2_ emissions avoided reach 13108 Mt over the period 2015–2050.
• As in the previous case, in the DAM-DEF approach — WE replaces all technologies — the DEF evolves as the energy mix does, which is especially relevant in the last years of the analysed period when the energy system is highly decarbonised so WE could replace other less-competitive RES. Overall, the annual CO_2_ emissions remain relatively constant showing a slight upward trend from 2030. They only increase by 88% from 116 Mt CO_2_ in 2015 to 218 Mt CO_2_ in 2050. The total CO_2_ emissions avoided reach 6581 Mt CO_2_ over the period 2015–2050.
• DHC-DEF and DAM-DEF represent the less and more conservative approaches respectively in terms of total CO_2_ emissions avoided by WE in the long run. The annual CO_2_ emissions avoided under these dynamic approaches increase from 116 to 203 Mt CO_2_ in 2015 up to 218–505 Mt CO_2_ in 2050 since WE penetration in the electricity system increases from almost 275 TWh to 980 TWh in the same time period. The total CO_2_ emissions avoided by WE in the period 2015–2050 range from almost 6600 to around 13100 Mt CO_2_.

### 4.4 Potential CO_2_ emissions abated in the EUMSs

**Fig. 10:**
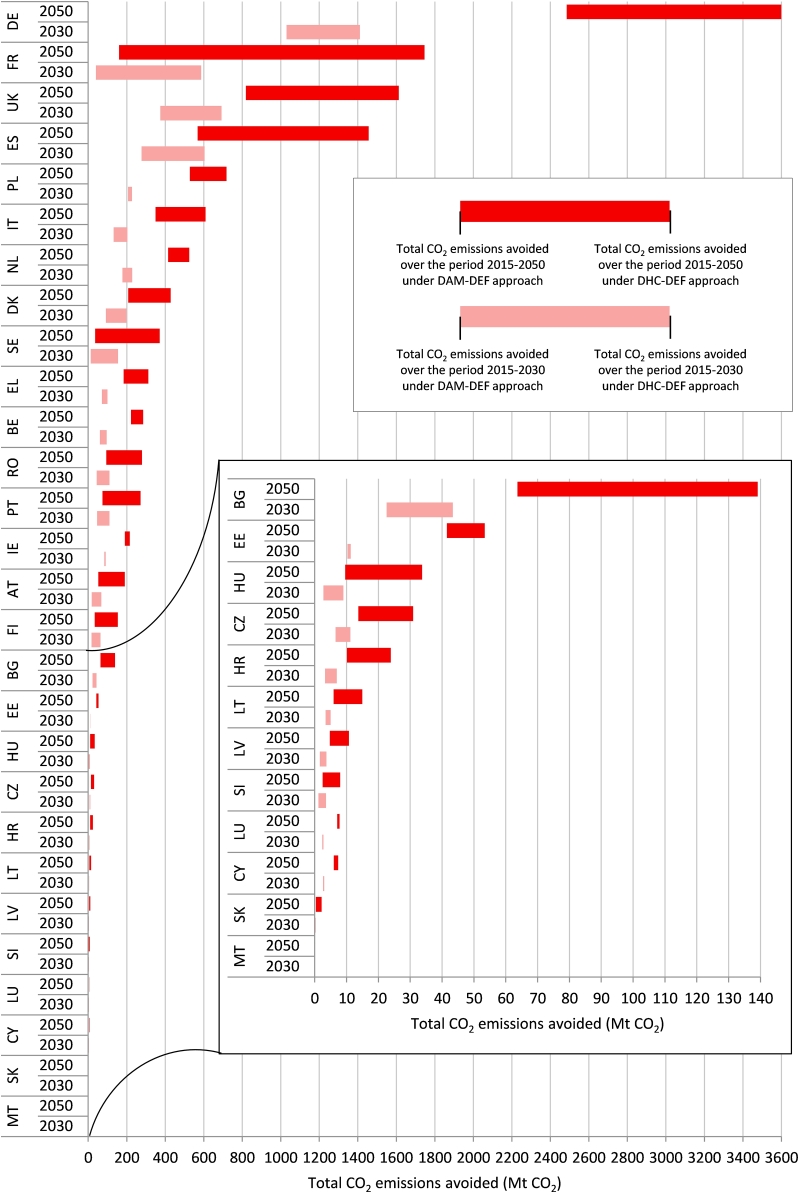
Range of total CO_2_ emissions avoided by WE according to the DAM-DEF (left value of each bar) and DHC-DEF (right value of each bar) approaches in 2030 and 2050.

Fig. 10 displays the range of total CO_2_ emissions abated by WE in the EUMSs over the period 2015–2030 (light red bars) and 2015–2050 (dark red bars) calculated under the DAM-DEF (represented by the left value of each bar) and the DHC-DEF (right value of each bar) approaches.

The EUMSs with a high penetration of gross wind electricity generation over the period 2015–2050 according to the *EU Reference Scenario 2016* rank in the top positions showing high potential CO_2_ emissions avoided. Nevertheless, in these EUMSs the total CO_2_ emissions abated show a high level of uncertainty when the range of values between the DAM-DEF and the DHC-DEF approaches is more expanded, *i.e.,* the bar is wider. The energy mix of these EUMSs shows two distinctive features: (i) the most polluting high-carbon technologies (mainly coal and lignite) have a significant role among the fossil fuel generation thus increasing the total CO_2_ emissions abated by WE under the DHC-DEF approach and (ii) a high share of gross electricity generation comes from renewables and nuclear decreasing the total CO_2_ emissions abated under the DAM-DEF approach.

As an example, the *EU Reference Scenario 2016* estimates Germany and France to show (i) a similar total gross electricity generation up to 2050 and (ii) wind generation to reach 196 TWh and 171 TWh respectively in 2050. In spite of these similarities, Germany and France show a different range of total CO_2_ emissions under the DAM-DEF and DHC-DEF approaches for two reasons. On the one hand, both countries are expected to decrease the fossil fuel generation over the period 2015–2050 but showing an opposite trend: coal and lignite electricity generation will dominate the fossil fuel production in Germany whilst France will shut down all coal-fired power plants by 2023. As a result, the total CO_2_ emissions displaced in Germany are significantly higher compared to France under the DHC-DEF approach. On the other hand, France is expected to show a high penetration of nuclear generation. Consequently, the total CO_2_ emissions are very low under the DAM-DEF approach.

### 4.5 Discussion

The assumptions on which technologies may potentially be displaced by wind energy generation depend considerably on the operating principles of the power system. Under current conditions, the operating margin is a widely accepted method in the literature to assess the CO_2_ abatement potential of wind energy: The most polluting technologies are usually displaced by wind energy according to the dispatch and schedule resulting from the bidding process of the current electricity markets. For this reason, most existing works that use a displacement estimation method to estimate the potential CO_2_ emissions avoided by wind energy are based on a fixed or static displacement emission factor corresponding to high-carbon generation displaced.

This approach may be adequate in the present but not in the long run when the energy mix of most countries becomes more and more decarbonised as promoted by the energy and climate change policies. On the one hand, the role of high-carbon generation will become more marginal and dominated by natural gas. Thus, wind energy is expected to replace different types of high-carbon technologies and in a different proportion compared to the current situation. On the other hand, the share of renewable energy sources will strongly increase. Consequently, wind energy could displace other less-competitive renewable energy generation.

According to the numerical results, the range of potential CO_2_ emissions abated by WE based on the proposed approaches (DAM-DEF and DHC-DEF) evolves from 116 to 203 Mt CO_2_ in 2015 up to 218–505 Mt CO_2_ in 2050 (see Fig. 8). The upper values result from the DHC-DEF and the lower values derive from the DAM-DEF.

As can be observed in Fig. 8, the approaches used in the literature (NG-DEF and HC-DEF) may underestimate the potential CO_2_ emissions abatement up to 2035 since the evolution of the high-carbon technologies displaced by WE is ignored. On the contrary, the HC-DEF approach may overestimate the emissions from 2035 onwards since the replacement of the most polluting high-carbon technologies by other lower carbon intensive technologies such as natural gas in the energy mix of most of EUMSs is not contemplated. Consequently, the DHC-DEF outperforms the displacement estimation approaches currently used by wind industry associations.

To reach a better estimation of the potential CO_2_ emissions displaced in the longer-term, the DAM-DEF approach could be suitable in those countries with an energy mix highly decarbonised since WE could replace not only high-carbon but also other low-carbon technologies.

Admittedly, both the DHC-DEF and the DAM-DEF approaches, which are based on dynamic emission factors computed on an annual basis, are characterised by several limitations, namely (i) they require *a priori* information such as the evolution of the energy mix, and thus they can be categorised as *ex-ante* methods; and (ii) they rely on simplifying assumptions of the electricity system since the impacts of the generating units' operation (*e.g.* ramping events, start-ups, shutdowns, among others) are internalised in the given evolution of the energy mix. Thus, a dispatch and unit commitment model may be suitable to compute the range of potential emissions. The open-source power system tool Dispa-SET (Quoilin et al., 2017), which is a dispatch and unit commitment model of the European electrical grid and mainly developed within the Joint Research Centre of the European Commission, may be used to perform such assessment. For each target year, daily simulations with hourly time steps can be run with and without wind generation in order to simulate the power system operation, and thus the annual CO_2_ emissions avoided by WE can be accurately computed. Notwithstanding, this method presents several drawbacks: (i) a commercial or open-source optimisation-based model is thus needed; (ii) the optimisation-based model requires excessive input data which are not readily available such as fuel prices, ramping rates, minimum up and down times, renewable energy projections, start-up and shutdown costs, storage-related data, and so on; (iii) it can be computationally expensive; and (iv) the results may be highly sensitive to assumptions on the input data (Holttinen et al., 2014).

## 5 Conclusions

This paper proposes a novel method to estimate the potential emissions avoided by wind energy based on two dynamic displacement emission factors that consider (i) the evolution of the energy mix and (ii) a possible displacement of less-competitive renewable energy sources in the long run. This improves upon the methods used by some wind industry associations such as WindEurope or the Global Wind Energy Council, which miscalculate the CO_2_ emissions averted by wind energy in the long-term in those energy systems with a high penetration of this technology as well as other RES and low-carbon intensive gas generation. In addition, this is the first manuscript that estimates the CO_2_ emissions avoided by wind energy in the European Union by using a dynamic displacement estimation approach.

This method is validated in the European Union energy mix over the period 2015–2050. The total CO_2_ abatement potential of wind energy could range from around 13100 Mt CO_2_ (if wind energy only displaces high-carbon technologies) to about 6600 Mt CO_2_ (if wind energy also displaces other low-carbon technologies) in such period. These figures mean around 4 and 2 times, respectively, the annual CO_2_ emissions of the energy sector in the EU in 2015. These values represent an upper and a lower bound compared to the results from approaches commonly used in the literature. Consequently, they may represent the less and more conservative effect of wind energy on the decarbonisation of the European energy system.

The method and results presented in this paper may be of interest to other researchers, practitioners, and policy makers since: (i) they allow assessing the contribution of wind energy to GHG abatement within the EU electricity system in the long-term, and (ii) they may help define a strategic energy planning with an adequate penetration of the different technologies that guarantees to meet the EU decarbonisation targets. These new approaches are based on the dynamics of the structure and composition of the electricity system, which need to be considered to properly evaluate which technologies may be actually displaced by new additions of wind energy generation.

The main limitations of the proposed approach are: (i) it is an *ex-ante* method, and (ii) it relies on simplifying assumptions of the electricity system. Therefore, the method provides an estimation of the CO_2_ emissions abated by wind energy given a generation energy mix. Finally, ongoing work will be devoted to computing the range of potential emissions by using an optimisation-based approach.

## Footnotes

2 According to the United States Environmental Protection Agency (https://www.epa.gov/ghgemissions/sources-greenhouse-gas-emissions), ‘*Carbon dioxide (CO*_*2*_*) makes up the vast majority of greenhouse gas emissions from the* [electricity] *sector*’. Therefore, this paper is focused on the CO_2_ emissions, as similarly done in other works in the technical literature (see Section 2).
